# 3D micro/nano hydrogel structures fabricated by two-photon polymerization for biomedical applications

**DOI:** 10.3389/fbioe.2024.1339450

**Published:** 2024-02-16

**Authors:** Hongxun Fu, Baojun Yu

**Affiliations:** Key Laboratory of Micro/Nano and Ultra-precision Manufacturing, School of Mechatronic Engineering, Changchun University of Technology, Changchun, Jilin, China

**Keywords:** micro/nano structures, two-photon polymerization, GelMA, biocompatibility, hydrogels

## Abstract

Hydrogels are three-dimensional natural or synthetic cross-linked networks composed of polymer chains formed by hydrophilic monomers. Due to the ability to simulate many properties of natural extracellular matrix, hydrogels have been widely used in the biomedical field. Hydrogels can be obtained through a variety of polymerization strategies such as heating and redox. However, photochemistry is one of the most interesting methods for researchers in this field. Gelatin-methacryloyl (GelMA) inherits the biological activity of gelatin and has become one of the gold standards in the field of biomaterials. GelMA, as a photopolymerizable hydrogel precursor, can be used to fabricate 3D porous structures for biomedical applications through two-photon polymerization. We report a new formulation of GelMA-based photoresist and used it to manufacture a series of two-photon polymerization structures, with a maximum resolution less than 120 nm. The influence of process parameters on 3D structures manufacturing is studied by adjusting the scanning speed, laser power, and layer spacing values in two-photon polymerization processing. *In vitro* biological tests show that the 3D hydrogel produced by two-photon polymerization in this paper is biocompatible and suitable for MC3T3-E1 cell.

## 1 Introduction

Hydrogels are three-dimensional (3D) natural or synthetic cross-linked networks composed of polymer chains formed by hydrophilic monomers (Mikos et al., 2006; Qin et al., 2014). Due to the ability to simulate many properties of natural extracellular matrix, hydrogels have been widely used in biomedical fields, such as drug delivery and tissue engineering (Vu et al., 2015; Bello, et al., 2020; Tianpeng et al., 2021; Wang et al., 2023; Fu et al., 2022). Hydrogels can be obtained through a variety of polymerization strategies such as heating and redox, however, photochemistry is one of the most interesting method for researchers in this field (Qin et al., 2014). Photoinitiator (PI) molecules in photosensitive hydrogel precursors absorb photons to produce free radicals, and then trigger free radical polymerization to form cross-linked hydrogel networks. The absorption of photons by PIs can be single photon or multi photons (Ovsianikov et al., 2014). Compared with traditional polymerization methods, it has many advantages, such as fast reaction speed, spatiotemporal controllability of the polymerization process, operability at physiological temperatures, etc (Qin et al., 2014).

Two photon polymerization (TPP) is based on optical nonlinear absorption (usually near-infrared femtosecond laser) to induce polymerization or crosslinking in photosensitive materials (LaFratta et al., 2007; Xing et al., 2015). When femtosecond laser is tightly focused into the material, a PI molecule absorbs two photons simultaneously and are excited to induce local free radical polymerization within the focus volume. It breaks away from the layer by layer paradigm and achieves true 3D arbitrary structure writing (Jing et al., 2022). Benefiting from the probability of two-photon absorption (TPA) proportional to the square of light intensity, and threshold characteristics of the polymerization process, TPP can fabricate structures with spatial resolution lower than the diffraction limit (Nguyen and Narayan, 2017; Paun et al., 2018; Parkatzidis et al., 2019). Correspondingly, studying the response of cell behavior (proliferation, differentiation, migration, and adhesion) to the physical, chemical, and biological characteristics of the surrounding environment at a subcellular scale (1–10 μm) and in well-defined high-resolution 3D structures has become a widespread consensus in the biomedical field (Tibbitt and Anseth, 2009; Raimondi et al., 2012; Akhmanova et al., 2015; Vu et al., 2015; Barin et al., 2022). Compared with traditional biomedical scaffold manufacturing technology, TPP has unique application advantages, mainly reflected in the spatial resolution of the structures and accurate CAD model replication. For example, using techniques such as phase separation, freeze-drying, and electrospinning to fabricate tissue engineering scaffolds cannot be compared with similar scaffolds manufactured by TPP in terms of precise geometric definition or spatial resolution (Accardo et al., 2020; Jing et al., 2022).

Gelatin is the main component of mammalian natural extracellular matrix, and the tripeptide arginine-glycine-aspartic acid (RGD) contained in its protein skeleton contributes to excellent cellular mutual activity. The gelatin-methacryloyl (GelMA) formed by the reaction of the primary amine of hydroxylysine, lysine and ornithine with methacrylic anhydride inherits the biological activity of gelatin. Therefore, GelMA has become one of the gold standards in the field of biomaterials (Van Hoorick et al., 2019; Xiang and Cui, 2021).

Previous studies have shown that GelMA, as a polymerizable hydrogel precursor, can be developed as porous scaffolds for cell inoculation through TPP (Ovsianikov et al., 2011a; Ovsianikov et al., 2011b; Prina et al., 2020). However, due to poor mechanical performance and swelling, it is difficult for the scaffolds to exceed sub-millimeter resolution even when manufactured with TPP at relatively high concentrations of GelMA (20 wt%) (Ovsianikov et al., 2011b). Some strategies are used to improve mechanical performance. For example, manufacturing structures on supports made of stronger materials or further methacrylylation of carboxylic acids present in GelMA to increase the number of photopolymerizable functionalities (Engelhardt et al., 2011; Wang et al., 2018; Wang et al., 2018). These methods either destroy the material properties of the structures or require complex chemical reactions. Particularly, TPP scaffolds with sub-micron resolution made from photosensitive solutions composed of GelMA and PEGDA with a degree of substitution (DS) of approximately 70% have been reported (Brigo et al., 2017). PEGDA is a kind of biocompatible synthetic photosensitive material, which has been manufactured into 3D hydrogel scaffolds with truly independent properties through TPP to allow the effective colonization of neuron cell line neuro2A (Accardo et al., 2018). However, due to issues with the strength of polymeric materials, the self-supporting ability of the scaffolds is insufficient, making the manufacturing of subcellular scale non-deformable 3D structures still a challenge. A recent paper reported the use of ruthenium complexes as photo-activators in TPP engineered gelatin-collagen matrixes. The GelMA used had a DS of 80% and a concentration of 20% (w/v). However, the photosensitive solution exhibited excessively high viscosity at room temperature, necessitating the entire process to be conducted at 40°C. Furthermore, the fabricated structures only had a resolution of approximately 10 μm and demonstrated noticeable swelling and deformation in the liquid environment (Van der Sanden et al., 2021).

In this article, the GelMA based photoresist was optimized by increasing the DS and adding crosslinking agents to significantly enhance the strength of the TPP structures. The preparation process is simple. Specifically, various 3D porous structures were manufactured using Poly (ethylene glycol) diacrylate PEGDA with a molecular weight of 400 Da as the crosslinking agent for GelMA of DS220 and P2CK as a two-photon specific initiator. To investigate the effects of processing parameters on the integrity and pore connectivity of TPP structures, different laser powers, scanning speeds, and layer spacing were used to fabricated 3D porous structures. Furthermore, the developed photoresist has to retain its favorable cell interactivity to remain suitable for biomedical purposes. Therefore, *in vitro* biological tests were performed on the structures.

## 2 Materials and methods

### 2.1 Synthesis of TPP- special photoinitiator

Owing its facile synthesis, superior water solubility and high initiation efficiency {[i.e., two photon absorption cross section (δ_TPA_): 140 GM at 800 nm]}, P2CK ((sodium 3,3′-(((1E, 1E′)-(2-oxocyclopentane-1,3-diylidene)bis (met hanylydiebe))bis (4,1-phenylene))bis (methylazanediyl))dipropanoate)) was synthesized as a TPP-special photoinitiator via protocol reported previously (Li et al., 2013). In brief, before precipitation in ethanol/hydrogen chloride solution, newly distilled cyclopentanone and benzaldehyde 3- [(4-formylphenyl)–methylamino] propionic acid were refluxed in NaOH solution for 6 h. This is a typical aldol condensation reaction. The product was vacuum dried after washing with cold methyl alcohol to obtain red powder.

### 2.2 Modification of gelatin

**FIGURE 1:**
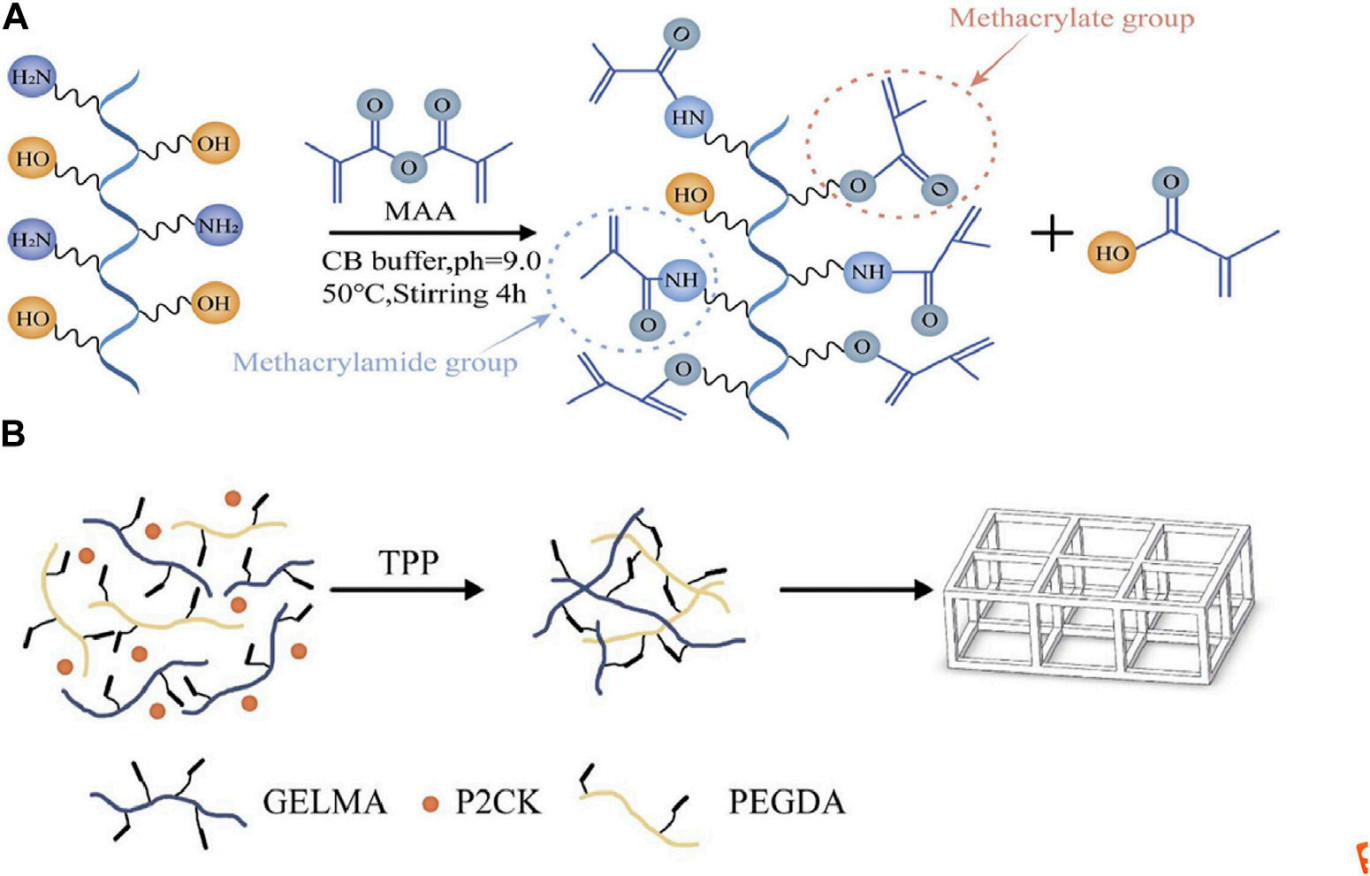
**(A)** Schematic illustration of GelMA (gelatin metacryloyl) synthesis. **(B)** The process scheme for TPP (two-photon polymerization) fabrication of the structures in this article.

We used the previously reported protocol to synthesize GelMA using a one-pot method (Shirahama, et al., 2016). Briefly, Type B gelatin with a bloom strength 250 was dissolved in 0.25 M CB buffer at a concentration of 10% (w/v) at 50°C, and the PH was adjusted with sodium hydroxide or hydrochloric acid. Afterwards, methacrylic anhydride (MAA) of 94% was added to the above solution. The reaction lasted for 4 h, and then the pH was adjusted to 7.4 to stop the reaction. This entire process was carried out under 500 rpm of magnetic stirring. After filtration, dialysis, and freeze-drying, the products were preserved at −20°C for further experiments (Figure 1A). The degree of substitution (DS) of GelMA was measured by ^1^H-NMR in deuterium oxide, and its calculation was based on previously reported literature (Zhu et al., 2019). Finally, GelMA with the DS of ∼220 was obtained.

### 2.3 Preparation of two-photon sensitive hydrogel solution (photoresist)

The photosensitive solution was formed by dissolving P2CK 5% (w/w), GelMA 70% (w/v), and PEGDA (average Mn 400) 5% (v/v) in PBS buffer, where PEGDA served as a crosslinking agent to further enhance the stiffness of the produced structures. The general process of photoresist preparation is to dissolve a certain amount of P2CK into PBS buffer until it is completely dissolved, and then add GelMA to the same centrifuge tube, and heat the centrifuge tube in a 37–50°C water bath for 1–2 h using a constant temperature water bath. After adding PEGDA, the centrifuge tube is water bathed at 37°C for 30–50 min and oscillated 3 times during this period. Finally, before storing it at 4°C for further use, the solution is filtered by 0.2 μm filter. The solution exhibits manageable flowability at room temperature. Figure 1B shows a brief process of TPP fabrication for the prepared photoresist.

### 2.4 Photorheology monitoring of photoresist photo-crosslinking reaction

Prior to TPP processing, the photo-reactivity of the photoresist was measured on a Anton-Paar MCR302 photorheometer, which possesses a parallel plate geometry. To conduct this test, 200 μL of PBS solution containing 0.25% (w/v) LAP (an efficient UV initiator), DS220-GelMA 70% (w/v), and PEGDA 5% (v/v) were placed between the plates with a gap of 0.3 mm. As a control group, photosensitive solution containing LAP0.25% (w/v), DS90-GelMA 20% (w/v) was also prepared and tested. The specific parameters for rheological testing were, shear strain:1%, oscillation frequency: 1 Hz, temperature: 37°C, time: 300 s, interval Time: 1.5 s, point number: 200, UV illumination time: 120 s, UV intensity: 30 mW/m^2^.

### 2.5 Mechanical testing by nanoindentation

The nanoindentation test was conducted on MML’s NanoTest Vantage nanoindenter. The Berkovich pyramid diamond indenter is adopted, with an angle of 65.03° between the three faces of the indenter and the axis of the diamond pyramid, and an angle of 120° between the three faces. The blunt radius of the indenter is 100 nm with the spring constant of 5 N/m. The Young’s modulus of the indenter is 1,141 GPa, and the Poisson’s ratio is 0.07. The tip radius was calculated by calibrating fused silica prior to the experimental procedure. The experiment was conducted in aqueous (PBS) environment with an ambient temperature of 23°C. The Hertz model is used to calculate the Young’s modulus. The tested samples were 6 cubes with dimensions of 100 μm × 100 μm ×6 μm (length× width × height), fabricated with a constant scanning speed 400 μm/s and different laser powers 5 mW, 10 mW, 15 mW, 18 mW, 22 mW, and 25 mW. Each sample was tested at 5 points with a Poisson’s ratio of 0.4.

### 2.6 TPP of the photoresist

**FIGURE 2:**
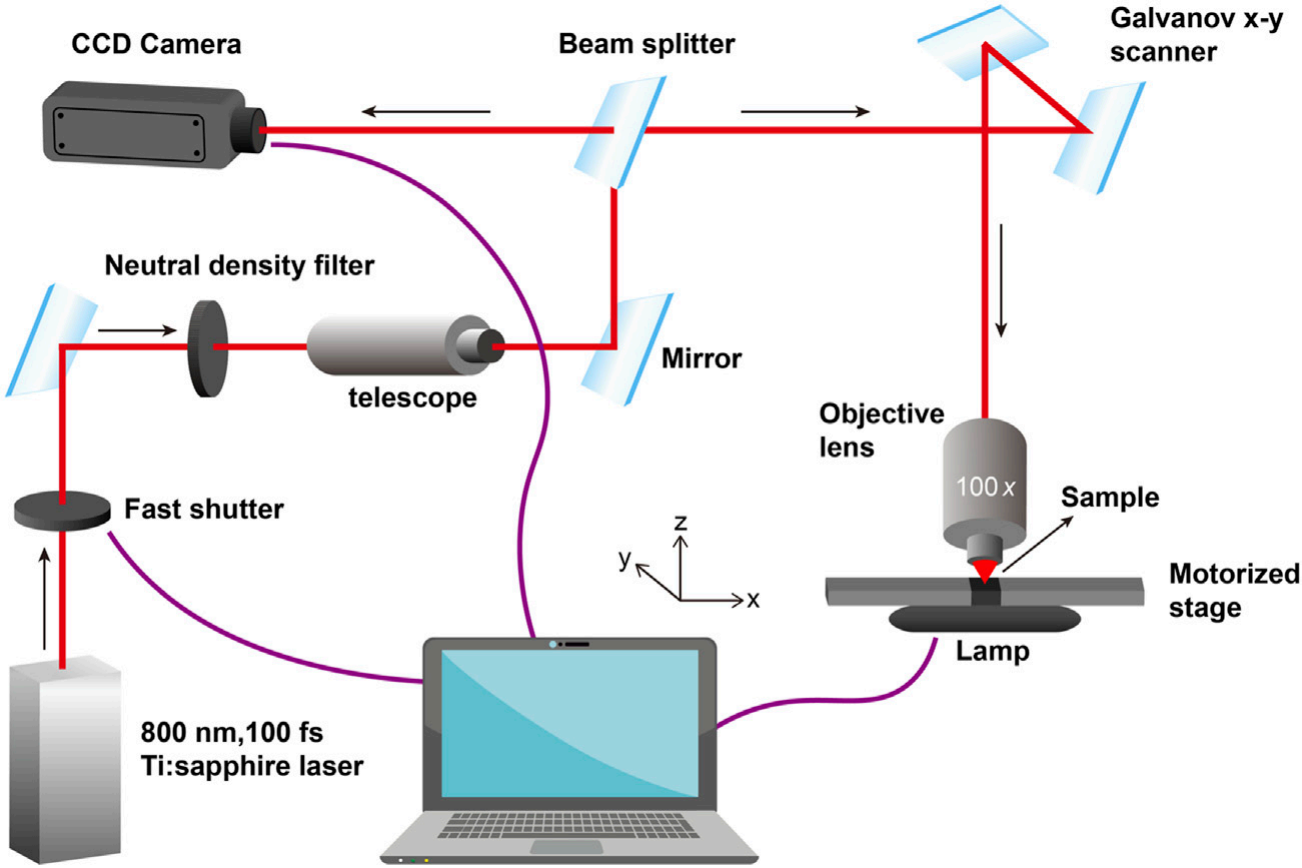
A typical experimental workstation for TPP in our lab.

The typical experimental workstation for TPP in our lab is given in Figure 2. A mode-locked Ti: sapphire oscillator with a repetition rate of 80 MHz, a wavelength of 800 nm, and a pulse duration of 100 fs, is used for TPP. The laser beam passing through the attenuator, beam expander, beam splitter and other optical components is tightly focused into the photopolymerizable materials with the oil-immersion objective lens (×100, NA = 1.3) filled with a refractive-index-matching oil (n_oil_ = 1.518). Photoresist is scanned by the laser focus in 3D space and polymerization occurs along the trace of the focus. After fabrication of the required structures, the samples must be developed to wash off the unpolymerized materials.

In this paper, the photoresist was placed in the cavity of a silicone elastic ring sandwiched between two cover glass slides to prevent solution evaporation. The bottom glass plate was silanized with 3-(trimethoxysilyl) propyl methacrylate to enhance the bonding strength between the polymer structures and the plate. The silanization solution consisted of deionized water (50% v/v), ethanol (48% v/v), glacial acetic acid (0.3% v/v), and 3- (trimethoxysilyl) propyl methacrylate (2% v/v), stirred for 15 min. The glass plates were pre-treated in a plasma cleaner for 10 min and then placed in the above solution for 30 min. After surface treatment, the glass plates were rinsed twice with deionized water and dried in a drying oven (50°C) for 2 h. After TPP processing, the structures were placed in PBS buffer at 37°C for more than 30 h to fully remove the unpolymerized materials, and then soaked in deionized water for further salt removal. Finally, the structures were obtained by freeze-drying.

To study the TPP threshold of the photoresist, lines were first processed at a fixed scanning speed (30 μm/s) but different laser powers (21.6 mW–1.8 mW). Based on the TPP threshold, we attempted to obtain the ultimate structural resolution of the photoresist.

The main factors affecting the connectivity and structural strength of three-dimensional porous structures are the scanning speed, laser power, and layer spacing of TPP. Therefore, the manufacturing of the 3D porous structures was carried out by fixing two of these parameters and changing the remaining one. The external dimension of the 3D porous structures’ CAD model is 44 μm × 44 μm × 24 μm (length × width × height), and the cross-sections of beams and columns are all 4 μm × 4 μm, that is, all square holes are 16 μm × 16 μm.

The polymer cubes with 1 mm × 1 mm × 5 μm (length× width × height) were fabricated for metabolic activity assay.

### 2.7 Characterization of structures fabricated via TPP

In order to perform SEM characterization, the structures fabricated on glass plates were first dehydrated in ethanol solutions of 70%, 80%, 90%, and three times 100% ethanol for 10 min. Then, after the 100% ethanol step, they were soaked in 100% hexamethyldisilazane (HMDS) for 3 min (Brigo et al., 2017). Prior to SEM imaging, the structures were subjected to four gold spray treatments, each lasting 1 min, with an Au/Pd layer thickness of approximately 10 nm. The structures were observed under a Tescan Vega3 SEM operating at 20 kV.

### 2.8 Metabolic activity assay

To test the effect of TPP processed polymers on cellular metabolic activity, polymer cubes were fabricated. Meanwhile, a new photosensitive solution was prepared using the biocompatible gold standard DS90-GelMA 20% (w/v) instead of DS220-GelMA in the photoresist mentioned above, and the same size structures were processed. The effect on MC3T3-E1 cells was determined using PrestoBlue assay according to the manufacturer’s instructions. In brief, the polymer cubes were transferred to a 24-well plate firstly. To sterilize the samples, UV−C irradiation (254 nm, 30 min) was applied prior to storage in the incubator overnight (5% carbon dioxide, 37°C) in appropriate medium. Next, all medium was aspirated from the samples, and 20,000 MC3T3-E1 cells was seeded per well. During further culturing, the appropriate cell medium was replaced every other day. The metabolic activity was tested using a PrestoBlue Cell Viability test (Life technologies) at specific time points (1, 2, 3, and 7 days). For the tests, PrestoBlue was diluted 1:10 with appropriate medium, and 500 μL of solution was applied per well followed by incubation for 1 h. In the presence of viable cells. From each well, 100 μL of solution was transferred to a 96-well plate for fluorescence measurements, and the remaining cell medium was aspirated and replaced by new appropriate medium followed by incubation. The fluorescence was measured with a plate reader (Synergy Bio-Tek, excitation 560 nm, emission 590 nm). After subtraction of sample blank (diluted PrestoBlue incubated for 1 h in appropriated medium), the different substrates were compared to each other and to the “dead cell” control (cells in 50% DMSO and 50% medium for 1 h). The fluorescence value obtained for the cells cultivated on tissue culture plastic (TCP) after 7 days of culture was considered as 100% viability. Next, all fluorescence values were normalized against this control and expressed relative to this 100% viability.

To verify the biocompatibility of materials further, MC3T3-E1 cells growth on various material surfaces were observed and recorded at day 1, day 2, day 3 and day 7 after cell isolation with density of 1 × 105 mL^−1^. The growth situation was observed through a bright field map. The cell counting method involved taking 10 fields of view (×40, ×10 eyepiece, magnification of 400) under a microscope for each group of structures at each time point. After counting the number of cells in each field, the average number was taken.

### 2.9 Statistical analysis

To evaluate the statistical significance of the obtained data, we first conducted an F-test on the two groups of variables to determine whether their variances were different. Next, conduct a Student’s *t*-test. When *p* < 0.05, the two values are considered significantly different.

## 3 Results and discussion

The main purpose of this article is to develop a novel photoresist composed mainly of natural derived materials to improve the stiffness of the products processed by TPP, thereby achieving higher spatial resolution and fully leveraging the technological advantages of TPP. The amount of photopolymerizable functionalities in GelMA with low DS (≤100) is limited, so we anticipate that increasing the DS while using a small amount of crosslinking agent would positively affect the cross-link density of the resulting photoresist.

### 3.1 Synthesis of GelMA with high degree of substitution

The side chains of different amino acids in gelatin contain different and easily reactive functional groups. For example, amine functional groups are present in the side chains of lysine, hydroxylysine, and ornithine, carboxylic acids are present in the side chains of glutamic acid and aspartic acid, and hydroxyl functional groups are present in the side chains of serine, threonine, and hydroxylysine. Currently, most commonly used modification strategies for GelMA with DS values less than 100%, including those mentioned in the introduction, involve the introduction of other functional groups using primary amines. However, in reality, hydroxyl groups can also exhibit nucleophilic behavior, thus participating in competition in the reaction. Research has shown that in the process of modifying gelatin to GelMA, hydroxyl functional groups were also observed to participate in the reaction when a 10 fold excess of MAA was added (Van Hoorick et al., 2019). These findings are of great significance for the modification and application of gelatin. As shown in Figure 1A, MAA reacts with primary amine functional groups to form methacrylamide groups, and reacts with hydroxyl functional groups to form methacrylate groups. This is the theoretical basis for the synthesis of high DS value GelMA in this study. In addition, different research reports have shown that 0.25MCB buffer is more reactive than 0.01 M PBS buffer for free amino groups (Shirahama, et al., 2016). This study achieved highly modified gelatin, and ^1^H-NMR measurements showed that the DS of GelMA was approximately 220%.

### 3.2 Determination of the mechanical properties of photoresist based on high DS GelMA

GelMA exhibits upper critical solution temperature (UCST) behavior, which means that the material forms collagen like triple helices below UCST, forming physical crosslinked networks (Van Hoorick et al., 2017). This makes 20% (w/v) DS90-GelMA close to its solubility limit at room temperature. However, the visual observation of the DS220-GelMA photosensitive solution prepared in this paper with a concentration of 70% (w/v) showed that it was still soluble at room temperature and did not form a physical gel. The high solubility can be attributed to higher side chain functionalization more effectively hindering the formation of the triple helix, which is consistent with the research results of some literature (Van Hoorick et al., 2017).

**FIGURE 3:**
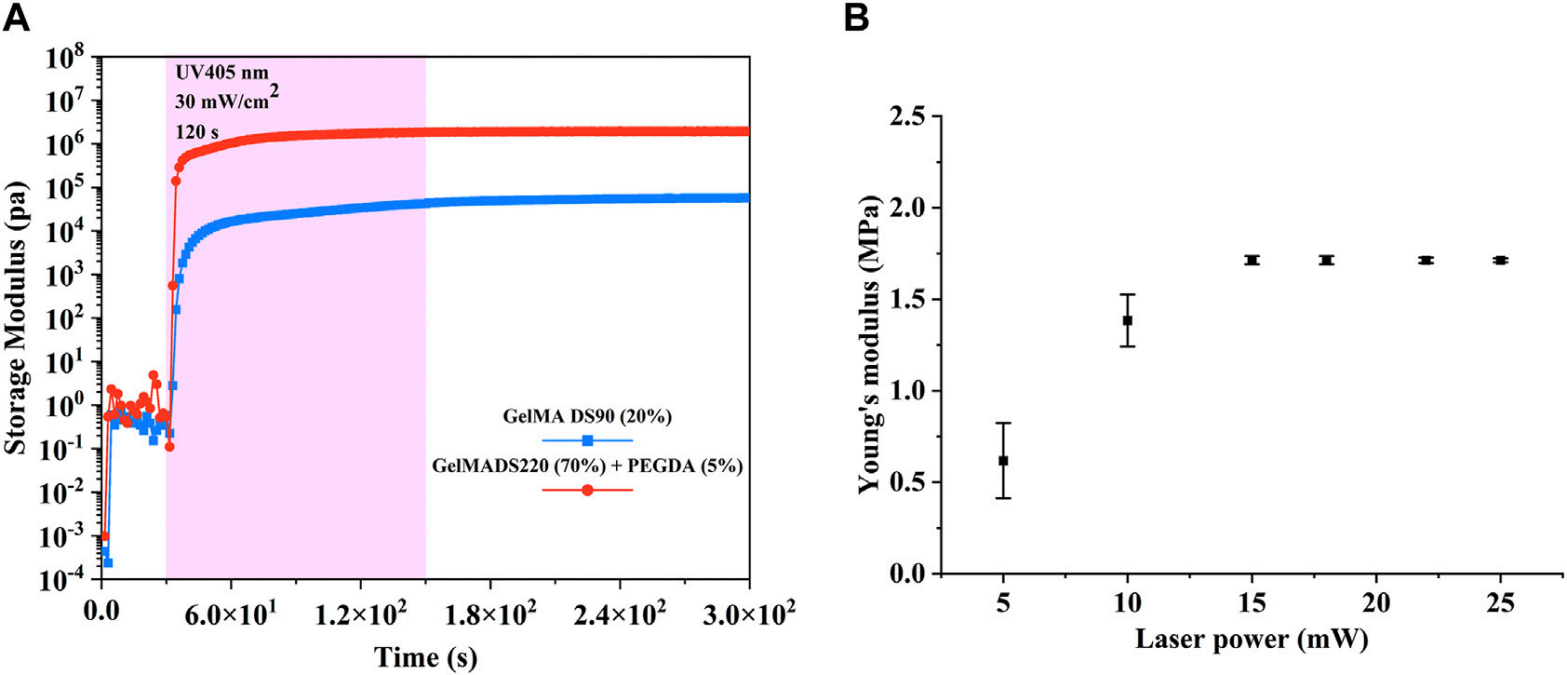
**(A)** Evolution of the storage modulus of 20% (w/v) DS90-GelMA and 70% (w/v) DS220-GelMA+5% (v/v) PEGDA during UV-A-induced cross-linking at 30 mW/cm^2^ as determined by rheology. **(B)** Young’s modulus of the cubic microstructures fabricated with different laser powers (testing in aqueous environment).

Photorheology experiments were conducted to determine whether the different amount of photopolymerizable functionalities for DS220-GelMA-based and DS220-GelMA-based photoresist has an influence on the final mechanical properties of the crosslinked hydrogels. Due to the fact that the storage modulus (G’) describes the elastic behavior of the materials and is related to the existing crosslinks, it was monitored for mechanical properties (Tytgat et al., 2019). As reported in the literature (Van Hoorick et al., 2017), an increase in double bond density in polymer precursors can lead to an increase in crosslink density, thereby enhancing the network of the polymer materials. It should be pointed out here that under the same conditions, the density of polymerizable functionalities enhances the cross-linking network, not just the quantity. Figure 3A shows the significant difference in G’ between the two photosensitive solutions we prepared. The G’ of DS220-GelMA/PEGDA is two orders of magnitude higher than that of DS90-GelMA, reaching 1.928 × 10^6^ Pa. To our knowledge, there are no similar tests of natural derived photopolymer materials that can demonstrate such high storage modulus. Besides, the slope of G’ also indicates that the photoactivity of high concentration and high DS photosensitive solutions is better than that of low ones (Figure 3A). The results of photorheological monitoring are consistent with expectations, that is, improving the mechanical properties of polymer materials by increasing the concentration of polymerizable double bonds.

From Figure 3B, we can see that the Young’s modulus at power 5 mW and 10 mW is significantly different from that at higher power, due to the insufficient number of free radicals caused by lower laser intensity and subsequent insufficient polymerization in the exposure area. When the energy per unit area increases, more free radicals are generated to increase the crosslinking density of the photoresist. Therefore, the Young’s modulus significantly increases when the laser power exceeds 15 mW. The increase in the Young’s modulus of materials helps to increase the stiffness of the structures. However, when sufficient polymerization free radicals are generated, further increase of free radicals is at least not beneficial, so the Young’s modulus remains basically unchanged when the laser power is between 15 mW and 25 mW. In addition, we can see that the Young’s modulus of the material in aqueous environment has reached ∼1.7 Mpa, which shows that our strategy of developing high-strength hydrogels based on natural derived materials is effective. The loading/unloading curves for nanoindentation testing can be found in the Supplementary Material. It should be pointed out here that although we have only carried out nanoindentation tests on polymers manufactured with different TPP powers, we can imagine that the modulus of hydrogels fabricated with different scanning speeds and layer spacing will also have similar phenomenon.

### 3.3 Structures fabricated by TPP

**FIGURE 4:**
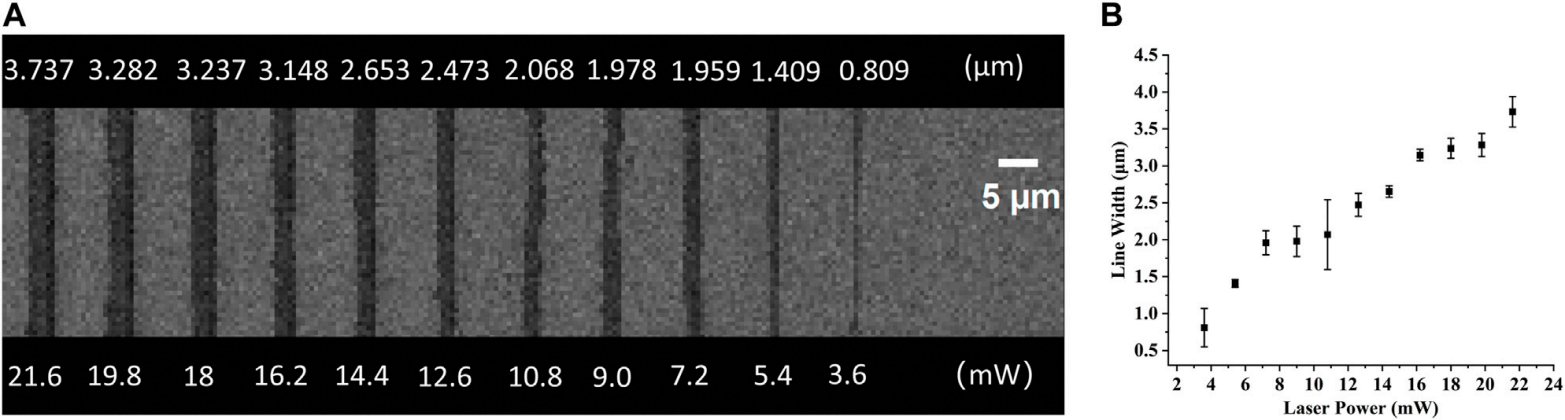
**(A)** Top view of the SEM images of lines fabricated with a single scan at a constant speed of 30 μm/s and varying powers. **(B)** The relationship of the laser power and the line width.

**FIGURE 5:**
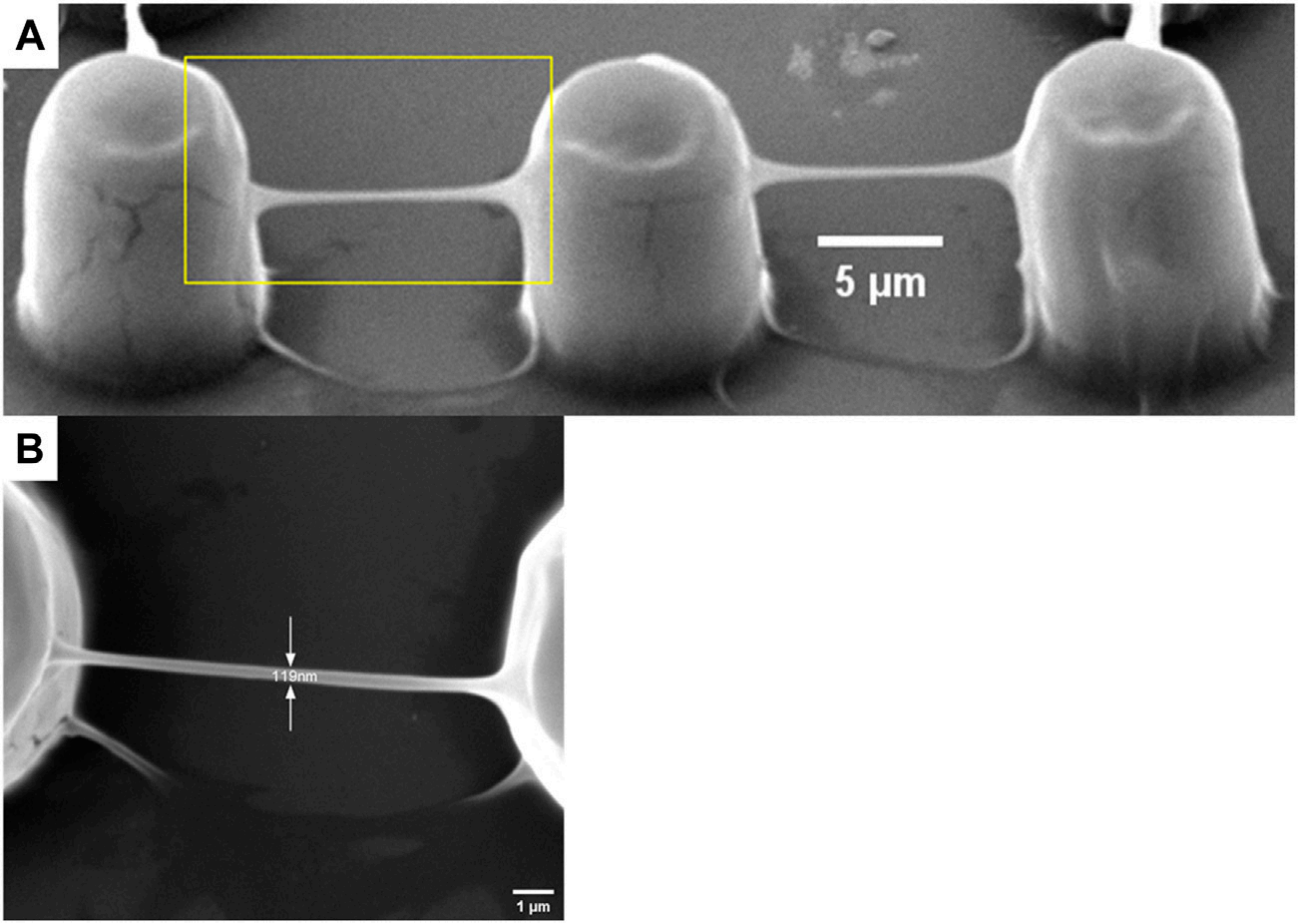
A polymer beam with a span of 10 μm processed at a scanning speed of 100 μm/s at the laser threshold of 1.8 mW. **(A)** oblique view and **(B)** top view.

In TPP, all entities processed are composed of voxels, including polymer lines. The voxel of TPP is an ellipsoid, so its short axis length is the width of the line in continuous scanning processing (Fischer and Wegener, 2012). In this article, when the laser power changed from 1.8 mW to 21.6 mW, polymer lines were fabricated with a single scan at a constant speed of 50 μm/s to determine the relationship between line width and laser power at that constant speed. As shown in Figure 4, as the laser power decreases, the line width also shows a decreasing trend, meaning that the resolution gradually improves. This is consistent with theoretical analysis and other literature reports (Gou et al., 2017). In the laser power of 3.6 mW, the line width is 809 nm (Figure 4). When the laser power is 21.6 mW, the line width is larger than 3,700 nm. In addition, we can see from Figure 4 that at 1.8 mW laser power, only one trace of polymer line was left, and its structure was damaged during the development process. As mentioned in a review (Jing et al., 2022), the laser threshold for two-photon absorption and two-photon aggregation are different, the latter refers to the laser intensity that enables the polymer structures to be retained after development. It is worth noting here that although single and multiple scans have no effect on line width, the strength of the structure and whether they can be retained in the subsequent development process cannot be ignored. A polymer beam with a span of 10 μm is processed at a scanning speed of 100 μm/s at the laser threshold of 1.8 mW, with a resolution of 119 μm, greatly exceeding the diffraction limit of the laser used (Figure 5). Such a high-resolution structure can be retained after development, benefiting from the high mechanical properties of the polymer materials we used.

**FIGURE 6:**
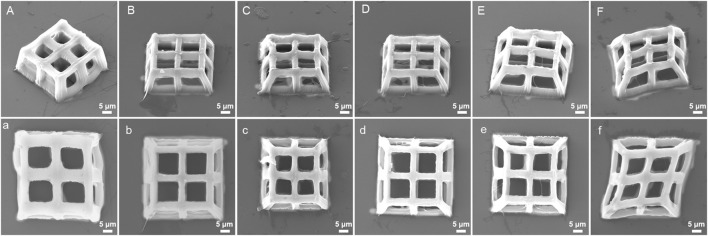
SEM images (oblique and top views) of 3D porous structures manufactured with constant scanning speed (400 μm/s) and layer spacing (500 nm) at six different laser powers: **(A)**, **(a)** 25 mW, **(B)**, **(b)** 22 mW, **(C)**, **(c)** 18 mW, **(D)**, **(d)** 15 mW, **(E)**, **(e)** 10 mW, and **(F)**, **(f)** 5 mW.

**FIGURE 7:**
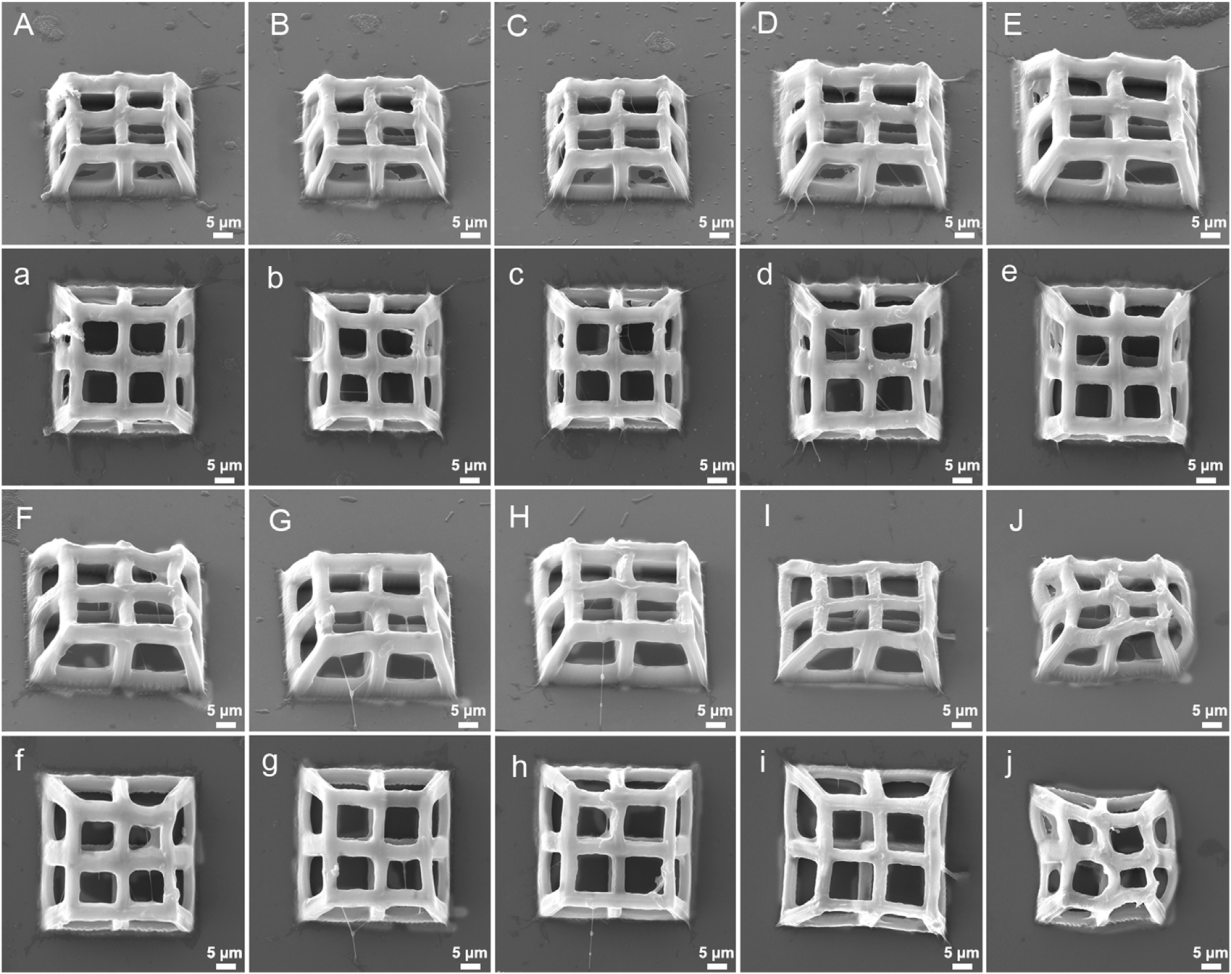
SEM images (oblique and top views) of 3D porous structures fabricatedwith constant laser power (18 mW) and layer spacing (500 nm) at varying scanning speed: **(A)**, **(a)** 400 μm/s, **(B)**, **(b)** 800 μm/s, **(C)**, **(c)** 1,200 μm/s, **(D)**, **(d)** 1,600 μm/s, **(E)**, **(e)** 2,000 μm/s, **(F)**, **(f)** 2,400 μm/s, **(G)**, **(g)** 2,800 μm/s, **(H)**, **(h)** 3,200 μm/s, **(I)**, **(i)** 3,600 μm/s, **(J)**, **(j)** 4,000 μm/s.

The stiffness of hydrogels produced by TPP is affected by laser power, scanning speed and layer distance (Gou et al., 2017). Therefore, we hypothesized that the integrity, stiffness, and pore connectivity of the 3D porous structures processed by TPP would also be affected by the above three parameters. At a constant scanning speed (400 μm/s) and layer distance (500 nm), the 3D porous structures were manufactured with varying laser power (Figure 6). Among the six laser powers of 25 mW, 22 mW, 18 mW, 15 mW, 10 mW, and 5 mW, when the power is high (25 mW, 22 mW, and 18 mW), there are polymer films left between the pores of the 3D structures produced (Figures 6A–C). When the power is low (5 mW), the structure collapses and deforms (Figure 6F), while at the powers of 15 mW and 10 mW, the structures’ integrity and pore connectivity are the best (Figures 6D, F). Besides, under constant laser power (18 mW) and layer distance (500 nm), different scanning speeds were used to process 3D porous structures. The scanning speeds were set from 400 μm/s to 4,000 μm/s, with an increment of 400 μm/s (Figure 7). As shown in Figures 7A–H, among the 10 scanning speeds, as the speed increases, the pores of the structure gradually become clear. However, when the speed exceeds 3,600 μm/s, the structures exhibit a lack of stiffness (Figures 7I, J). From the above experimental results, we can see that the influence of laser power and scanning speed on the manufacturing of TPP structures is significant, and the fundamental reason for this effect is the number of free radicals generated during the two-photon absorption process. When the scanning speed remains constant and the laser power changes, the lower the laser intensity, the less free radicals generated in the exposure area, resulting in insufficient polymerization of the photoresist and affecting the formation of the structures. Similarly, when the laser power is fixed and the scanning speed is changed, the faster the scanning speed, the shorter the exposure time, the insufficient density of the generated free radicals leads to insufficient crosslinking, thereby affecting the stiffness of the structures. However, if the laser intensity or exposure time is too large, the free radicals in the exposure area exceeds a certain density, the increase of voxel size and the diffusion of free radicals will have a negative impact on the resolution and connectivity of porous structures.

**FIGURE 8:**
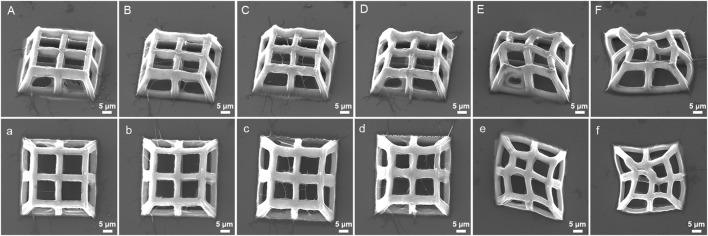
SEM images (oblique and top views) of 3D porous structures fabricated with constant laser power (18 mW) and scanning speed (400 μm/s) at varying layer spacing: **(A)**, **(a)** 100 nm, **(B)**, **(b)** 300 nm, **(C)**, **(c)** 500 nm, **(D)**, **(d)** 700 nm, **(E)**, **(e)** 900 nm, and **(F)**, **(f)** 1,100 nm.

The TPP processing strategy of constant scanning speed, laser power, and varying layer spacing was also used for the manufacturing of 3D porous structures (Figure 8). At 10 mW power and 400 μm/s scanning speed, the processed structures with layer spacing of 900 nm and 1,100 nm have low strength and severe deformation (Figures 8E, F), while excessively small layer spacing (100 nm) is not conducive to the clarity of pores (Figure 8A). When the layering distance is within a moderate range, there are no significant changes in the pore connectivity of the structures (Figures 8B–D). On the premise that other conditions remain unchanged, the layer spacing is closely related to the strength of the structure, that is, the smaller the layer spacing, the more stacking between the voxels, and the higher the polymerization density of the photoresist, *vice versa*. When the layer spacing reaches a certain level, the polymer layers will separate from each other, causing structural collapse.

Although it is beyond the research scope of this paper, it should be noted here that the 3D structure in the SEM pictures shows a certain degree of shrinkage, which is inevitable after the hydrogel absorbs a large amount of water and dries. If the structure processed by TPP is stored in liquid, shrinkage will not occur, as confirmed by the literature (Van Hoorick et al., 2017). In addition, from the neat lower edges of all structures, it can be seen that there is almost no swelling phenomenon in the TPP structure in this article, which is consistent with other literature reports (Van Hoorick et al., 2017).

### 3.4 Biocompatibility of 3D structures fabricated by TPP

**FIGURE 9:**
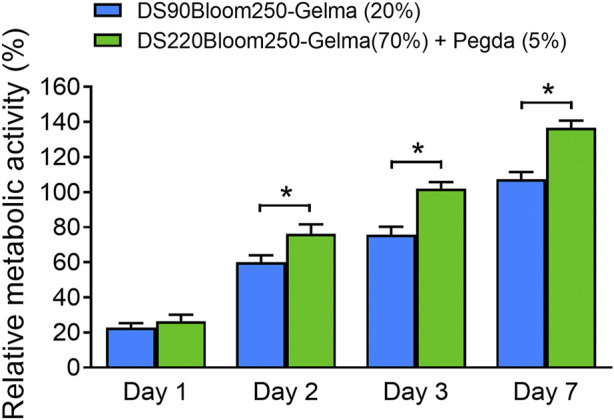
Presto blue assay was performed on structures made of two kinds of photoresist expressing the metabolic activity of MC3T3-E1 cell, *, *p* < 0.05.

**FIGURE 10:**
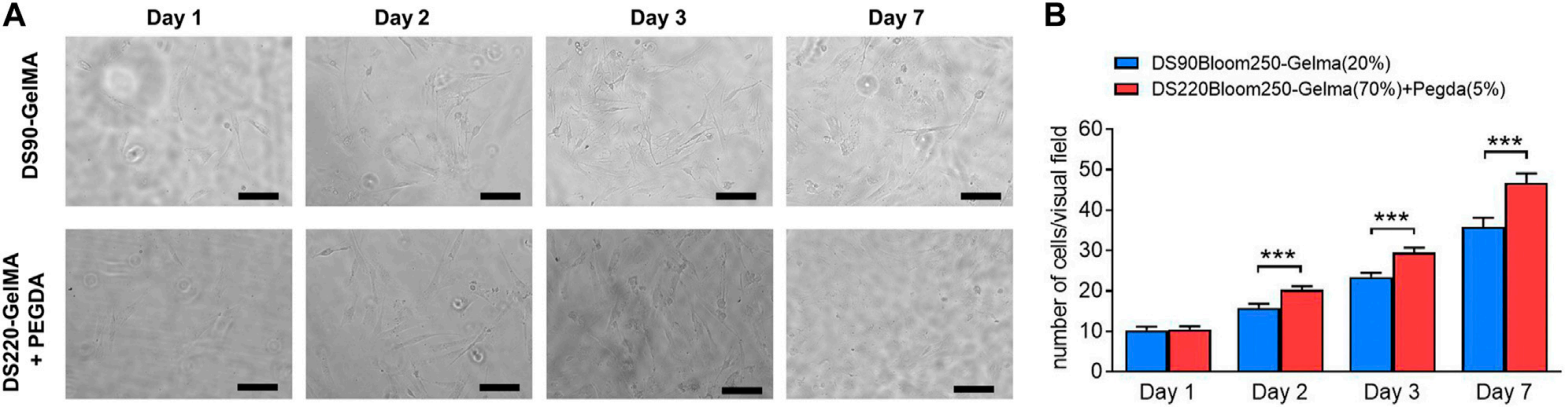
**(A)** The bright field map of MC3T3-E1 cells growth on structures, day 1, day 2, day 3 and day 7 respectively. The upper structures were fabricated by 20% (w/v) DS90-GelMA through TPP, while the lower structures were manufactured by 70% (w/v) DS220-GelMA+5% (v/v) PEGDA. Scale bars represent 100 μm. **(B)** Count statistical analysis chart of MC3T3-E1 cells growth on structures, day 1, day 2, day 3 and day 7 respectively, ***, *p* < 0.005.

Despite the favorable material properties, the developed photoresist has to retain its favorable cell interactivity to remain suitable for biomedical purposes. Therefore, *in vitro* biological tests were performed on structures fabricated by DS90-GelMA and DS220-GelMA photoresist using MC3T3-E1 cells. The metabolic activity of the cells was monitored at regular time points using a PrestoBlue assay. The results of the assays are depicted in Figure 9. Because a clear increase in metabolic activity is observed as a function of time, the cells can be considered healthy and proliferating on all substrates throughout the course of the experiment. In the performed assay, confluence was indeed reached between days 3 and 7 resulting in a plateau in metabolic activity, and all substrates exhibited a metabolic activity of >70% after 7 days of culture. As a consequence, both materials can be considered biocompatible and suitable for MC3T3-E1 cell. In addition, the results of bright field map in Figure 10A indicated that cells grown on the structures fabricated with DS220-Gelma/PEGDA, showed better cell extension and propagation, than cells grown on the structures prepared with DS90-GelMA. The number of cells on the DS220-GelMA/PEGDA structures significantly exceeded that on DS90-GelMA structures, after 3 days of culture, as shown in Figure 10B.

## 4 Conclusion

A novel hydrogel precursor photoresist based on GelMA for TPP has been prepared and used to manufacture 3D structures. In particular, the highly substituted GelMA used in the paper was obtained through a simple one pot synthesis method. The structures exhibited higher strength than previous types of GelMA-based photoresists. The effects of laser power, scanning speed, and layer spacing on the 3D structures manufactured in TPP have been investigated. The metabolic activity test showed that the 3D hydrogels possess satisfactory biocompatibility. The study in this article provides new ideas for the combination of TPP as a high-resolution additive manufacturing technology and GelMA as a promising natural derivative material for the biomedical applications, including drug delivery and tissue engineering.

## Data Availability

The original contributions presented in the study are included in the article/Supplementary Material, further inquiries can be directed to the corresponding author.
