# Supplementary figures and images for: 3D micro/nano hydrogel structures fabricated by two-photon polymerization for biomedical applications
Source: Front Bioeng Biotechnol. 2024 Feb 16;12:1339450. doi: 10.3389/fbioe.2024.1339450 (PMC10904474; doi:10.3389/fbioe.2024.1339450)

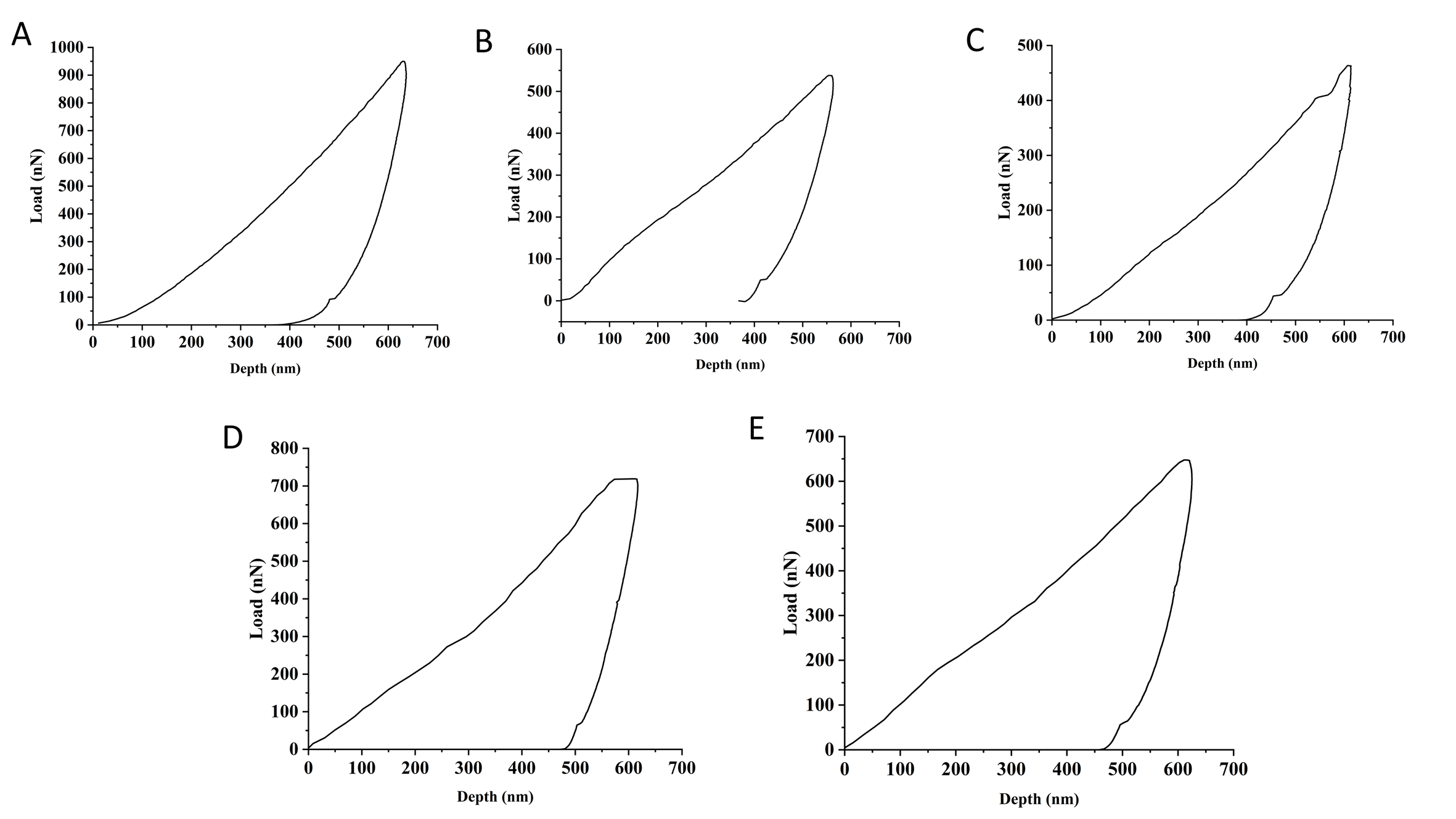

Supplement: Supplementary file 1 [file Image6.TIF]

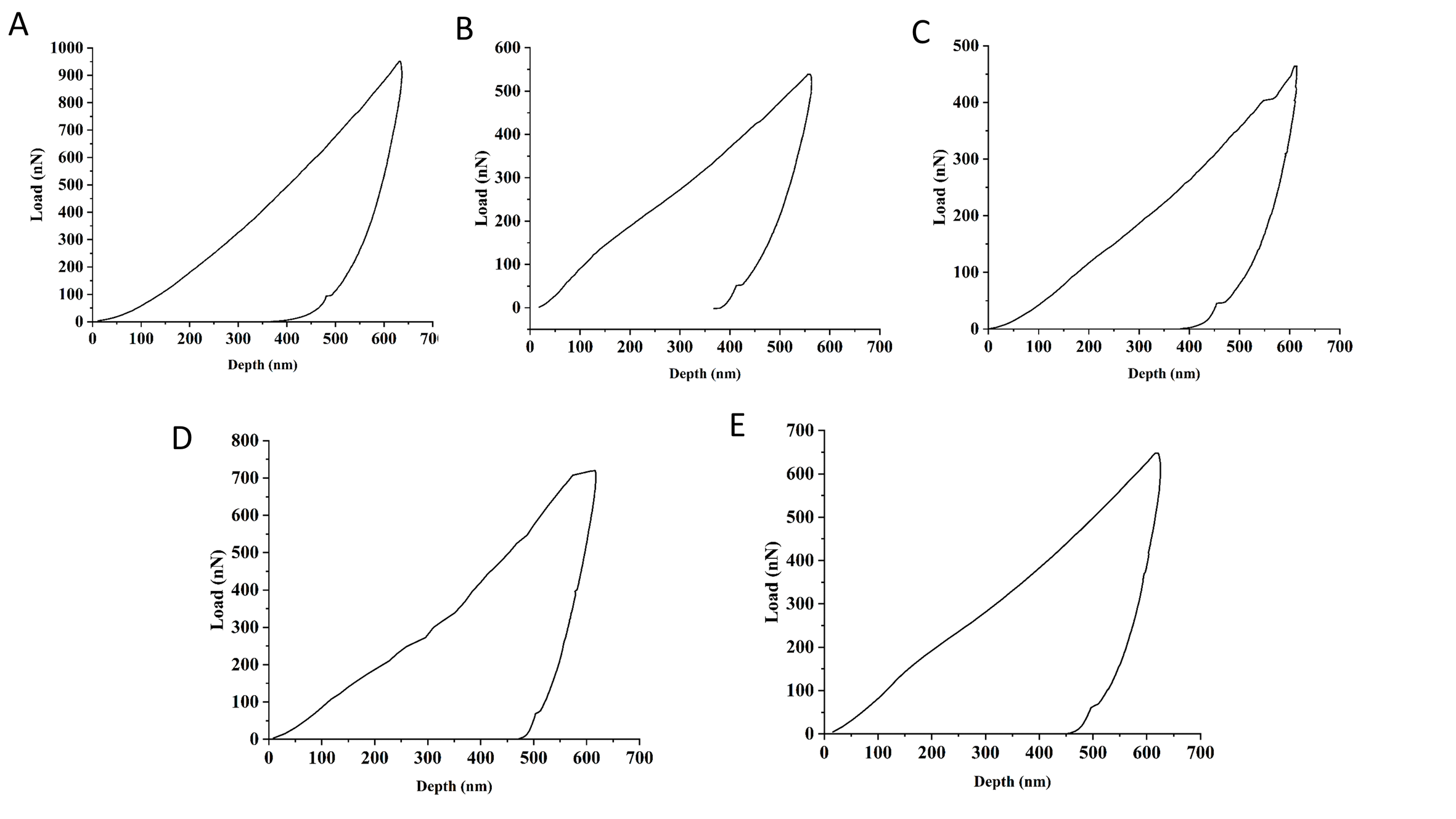

Supplement: Supplementary file 3 [file Image3.TIF]

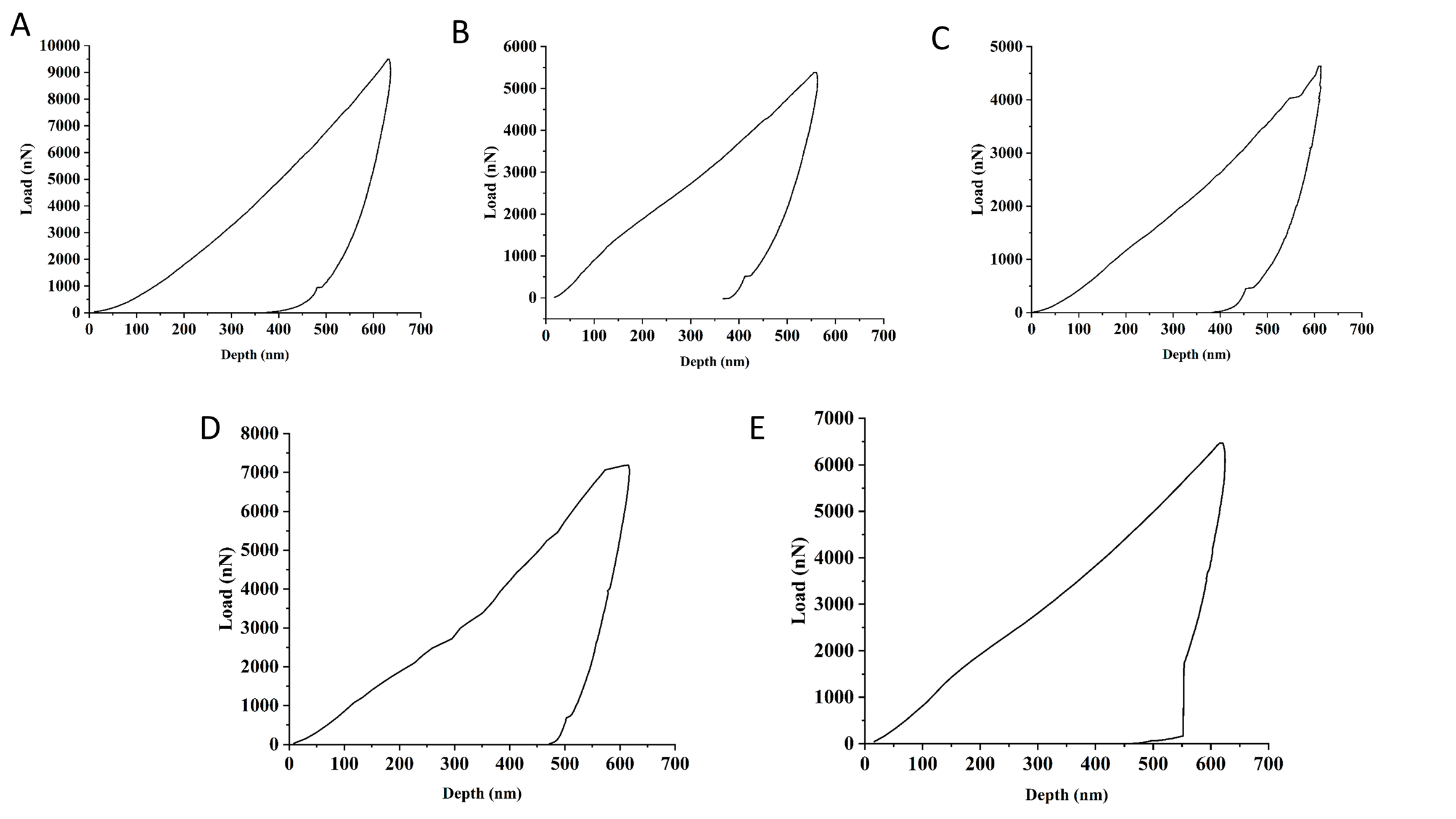

Supplement: Supplementary file 4 [file Image4.TIF]

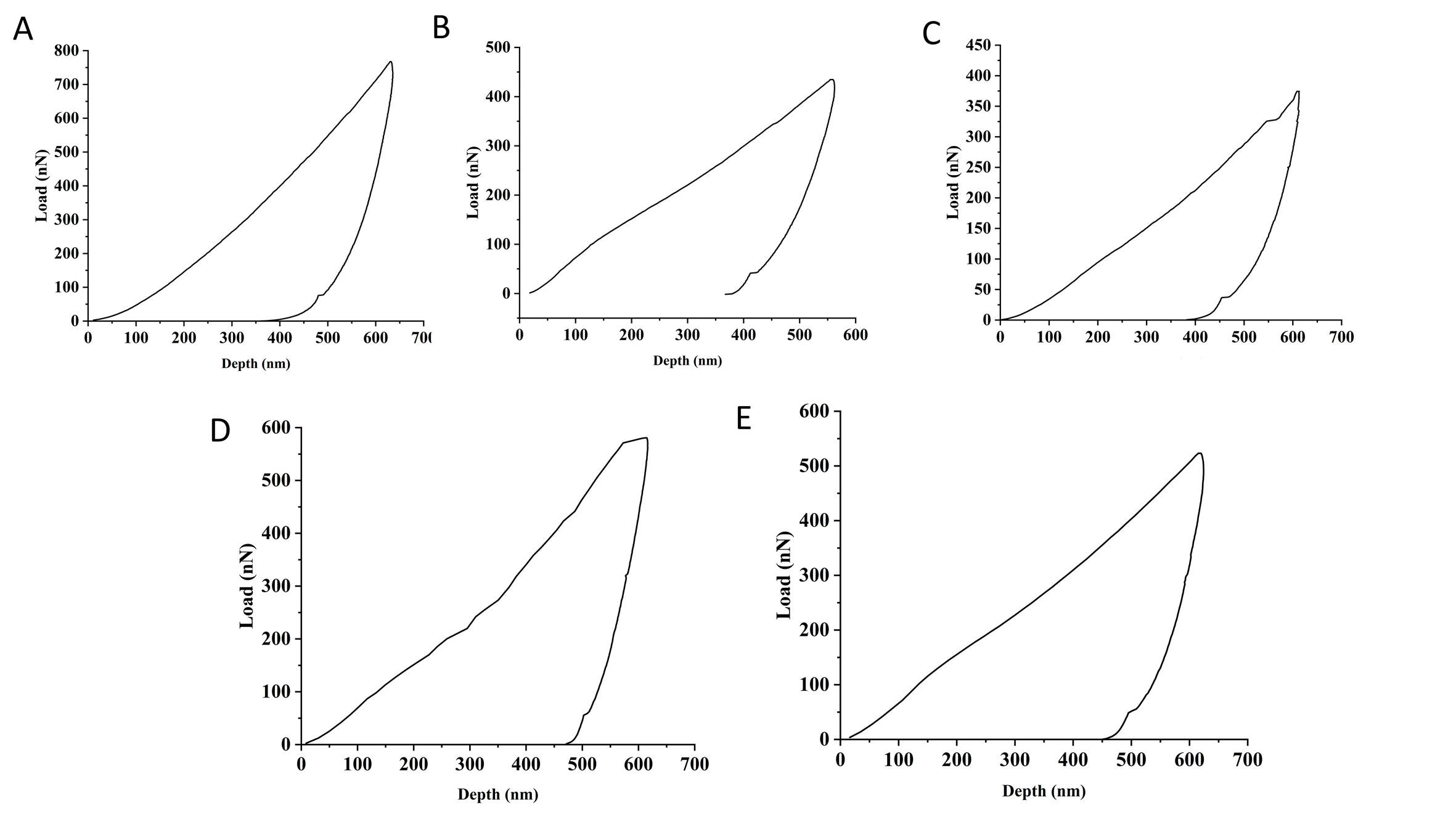

Supplement: Supplementary file 5 [file Image2.TIF]

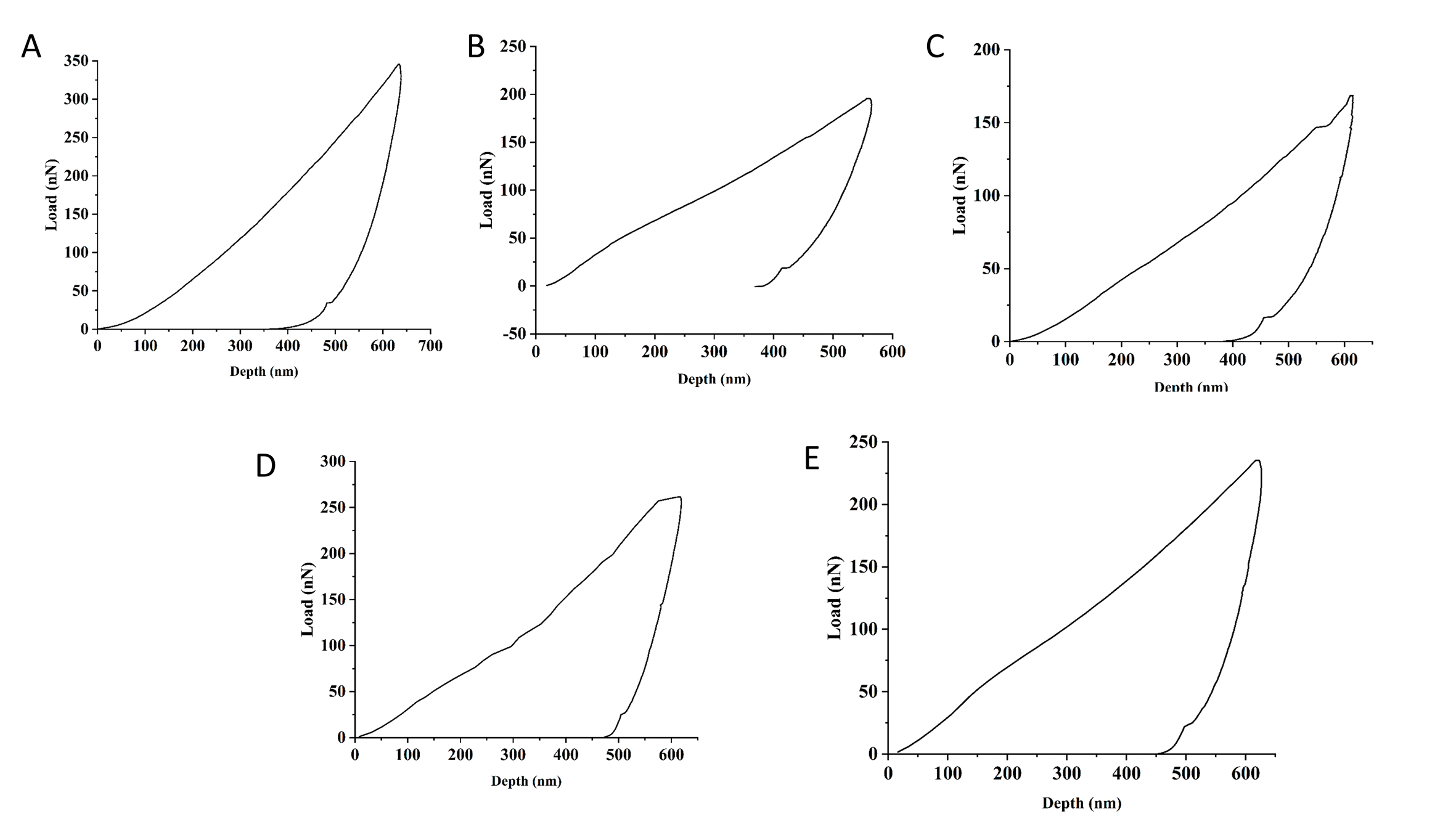

Supplement: Supplementary file 6 [file Image1.TIF]

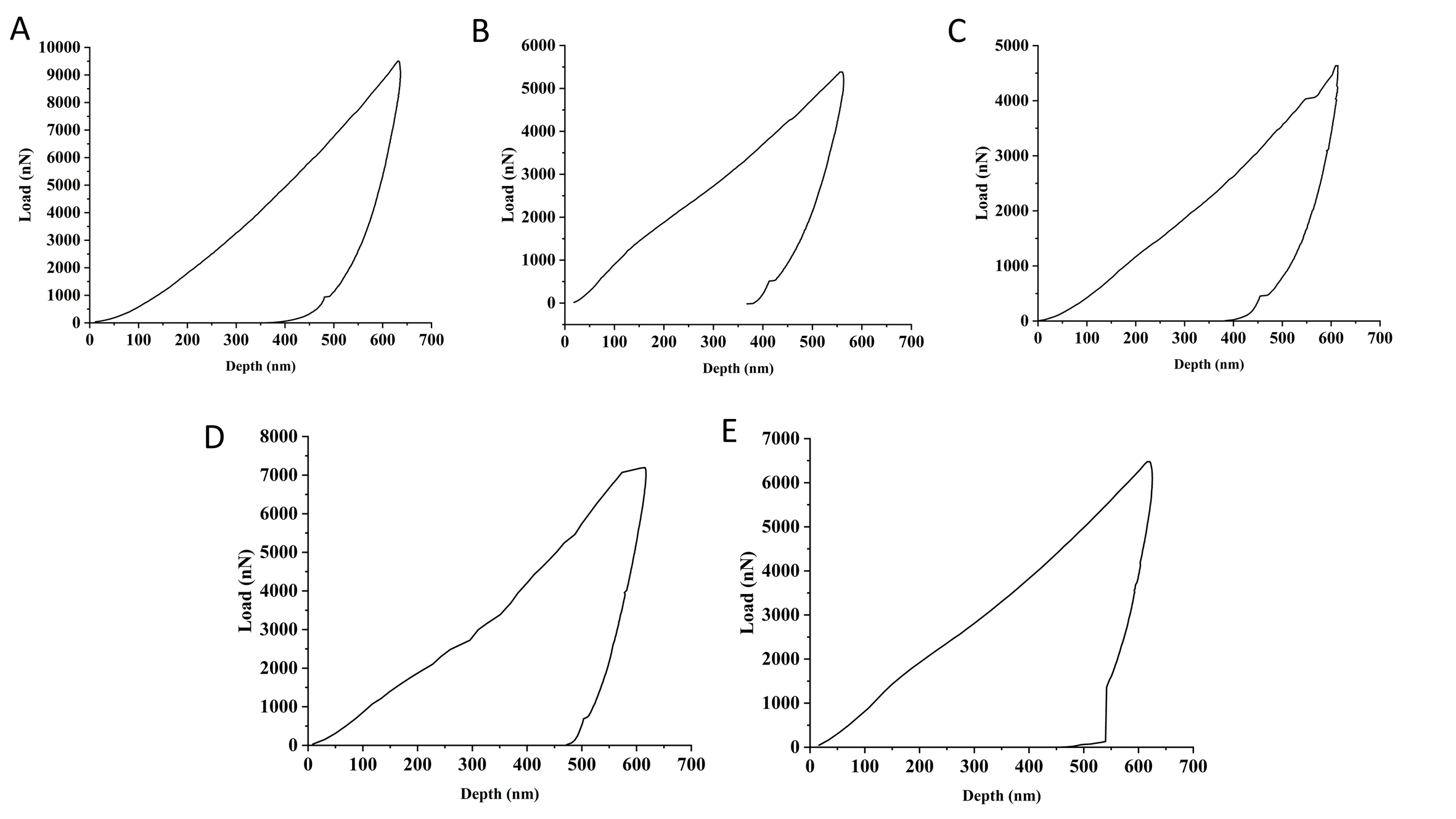

Supplement: Supplementary file 7 [file Image5.TIF]
