# Supplementary material for: 3D micro/nano hydrogel structures fabricated by two-photon polymerization for biomedical applications
Source: Front Bioeng Biotechnol. 2024 Feb 16;12:1339450. doi: 10.3389/fbioe.2024.1339450 (PMC10904474; doi:10.3389/fbioe.2024.1339450)
Supplement: Supplementary file 2 [file Table1.DOCX]

Supplementary Material

**Supplementary Figure 1. (**A) - (E), loading/unloading curve (5 points) of nanoindentation test of hydrogel fabricated at 400 μm/s scanning speed and 5 mW laser power.

**Supplementary Figure 2. (**A) - (E), loading/unloading curve (5 points) of nanoindentation test of hydrogel fabricated at 400 μm/s scanning speed and 10 mW laser power.

**Supplementary Figure 3. (**A) - (E), loading/unloading curve (5 points) of nanoindentation test of hydrogel fabricated at 400 μm/s scanning speed and 15 mW laser power.

**Supplementary Figure 4. (**A) - (E), loading/unloading curve (5 points) of nanoindentation test of hydrogel fabricated at 400 μm/s scanning speed and 18 mW laser power.

**Supplementary Figure 5. (**A) - (E), loading/unloading curve (5 points) of nanoindentation test of hydrogel fabricated at 400 μm/s scanning speed and 22 mW laser power.

**Supplementary Figure 6. (**A) - (E), loading/unloading curve (5 points) of nanoindentation test of hydrogel fabricated at 400 μm/s scanning speed and 25mW laser power.
